# Special Issue on Efficacy, Safety, and Immunogenicity of Vaccines Against Viruses: From Network Medicine to Clinical Experimentation

**DOI:** 10.3390/v18020165

**Published:** 2026-01-27

**Authors:** Pietro Hiram Guzzi, Jayanta Kumar Das, Marianna Milano

**Affiliations:** 1 Department of Surgical and Medical Sciences, University of Catanzaro, Viale Europa, 88100 Catanzaro, Italy; 2 Biomedical Research Center, National Institute on Aging, National Institutes of Health, Bethesda, MD 21224, USA; dasjayantakumar89@gmail.com; 3 Department of Experimental and Clinical Medicine, University of Catanzaro, 88100 Catanzaro, Italy; m.milano@unicz.it

In an era defined by the constant emergence of novel viral threats—from rapidly evolving SARS-CoV-2 variants to endemic pathogens such as influenza and orthohantaviruses—the scientific community is continually challenged to refine the tools and strategies that shape modern vaccinology. Vaccines remain one of the most effective public health interventions ever developed; yet, the increasing diversity of viral lineages, the complexity of immune responses, and the heterogeneity of human populations now demand an integrated and multidisciplinary approach. Traditional immunological assays alone are no longer sufficient to fully capture the nuances of vaccine-induced protection. Instead, contemporary vaccinology sits at the intersection of computational modeling, network medicine, and clinical experimentation.

This second edition of *Efficacy, Safety, and Immunogenicity of Vaccines against Viruses* builds upon the foundations of the first, expanding its scope to reflect the rapid scientific advances of recent years. Central to this volume is the recognition that immunity represents a dynamic and interconnected system: a network of molecular signals, cellular pathways, and population-level interactions. Understanding such complexity requires analytical frameworks capable of integrating diverse data sources and revealing patterns that may elude traditional methodologies.

The first contribution [1] in this collection demonstrates this shift through the development of an interpretability framework that combines machine learning and deep learning to analyze COVID-19 symptomatology and vaccine response. Employing SHAP values, LIME, counterfactual explanations, and gradient-based techniques, the authors uncover unexpected predictive features—illustrating how interpretable artificial intelligence can support clinical decision-making and refine public health strategies.

A second study [2] explores whether immune responses elicited by unrelated vaccines, such as tetanus toxoid or hepatitis B, may serve as baseline predictors of immunogenicity in an ALVAC-HIV and AIDSVAX B/E regimen. The detection of moderate but meaningful correlations offers a compelling starting point for new methodological perspectives in HIV vaccine research, suggesting that pre-existing immune signatures may inform the design and interpretation of future vaccine trials.

The third article [3] presents an immunoinformatics-driven approach to designing multiepitope peptide constructs targeting Puumala orthohantavirus. By identifying conserved regions with broad HLA coverage and demonstrating stable predicted interactions with Toll-like receptor 4, the authors highlight how computational pipelines can accelerate vaccine development, particularly for zoonotic pathogens where laboratory validation is challenging or slow.

A further contribution [4] addresses the growing concern of immune escape in emerging SARS-CoV-2 variants, evaluating how mutations in BA.2.86 and JN.1 affect known T-cell epitopes induced by ancestral and XBB-based vaccines. Although these variants alter a substantial number of epitopes, the study concludes that widespread loss of T-cell recognition is unlikely, underscoring the resilience of cellular immunity in maintaining vaccine protection over time.

Finally, the issue includes a comprehensive review of influenza vaccine immunogenicity [5], spanning animal models, human challenge studies, and evolving regulatory criteria. By contextualizing decades of research, the authors illustrate the ongoing difficulty of defining universal correlates of protection for a virus that continually adapts.

Collectively, the works in this Special Issue reaffirm that advancing vaccine science requires both innovation and integration. Through computational insight, experimental rigor, and interdisciplinary collaboration, these studies illuminate new pathways for understanding immunity and strengthening global preparedness.
